# A correlation analysis between arsenic and mercury resistance and the spread of antibiotic resistance genes in bacteria isolated from a highly contaminated brownfield

**DOI:** 10.3389/fmicb.2026.1873249

**Published:** 2026-08-27

**Authors:** Alexander Prosenkov, Daniel Sala Fernández, José Luis R. Gallego, Ana Isabel Peláez

**Affiliations:** 1 Area of Microbiology, Department of Functional Biology, Environmental Biogeochemistry and Raw Materials Group and IUBA, University of Oviedo, Oviedo, Spain; 2 INDUROT and Environmental Biogeochemistry and Raw Materials Group, Campus of Mieres, University of Oviedo, Mieres, Spain

**Keywords:** antibiotics, arsenic and mercury resistance, bacteria, biocides, brownfield site, mobile genomic elements

## Abstract

Seventy-four bacterial strains from El Terronal (Asturias, Spain), a brownfield highly contaminated with mercury and arsenic but without anthropogenic antibiotic influx, were isolated and characterized in culture. No correlation was found between resistance to the aforementioned metal(loid)s and to antibiotics and biocides as tested by agar plates and disk diffusion method, as the number of strains deemed resistant to any of the analyzed antibiotics and biocides was not significantly higher among those with the highest resistance to arsenic and/or mercury. Genome sequencing of 17 of the isolated strains revealed a great number and diversity of antibiotic resistance genes (ARGs), as well as genes related to arsenic and mercury resistance, some of which were located in mobile genetic elements (MGEs). However, most of the detected ARGs were not located in MGEs, and no genes responsible for metal(loid) and antibiotic resistance were found to share an MGE. No evidence was found that resistance to arsenic and mercury influenced the dissemination of ARGs in the studied strains, although the abundance of antibiotic resistance mechanisms related to the presence of RND-type (Resistance-Nodulation-Division) efflux pumps could potentially contribute to cross-resistance.

## 1 Introduction

There is a growing concern about the increasing presence of antibiotic resistance genes (ARGs) in the environment (water, manure, and soil) due to the widespread use and misuse of antibiotics in clinical practice, livestock farming, and other activities (Ahmed et al., 2024; Matheou et al., 2025). ARGs are mostly found in mobile genetic elements (MGEs) such as transposons, integrons, plasmids, extracellular DNA, and phages that can be transferred between strains of the same bacterial species and between different species (horizontal gene transfer, HGT, or lateral gene transfer, LGT), through mechanisms of conjugation, transduction, and natural transformation (Bobate et al., 2023). In this way, they can end up in pathogenic strains of clinical importance, with serious consequences for mortality rates associated with both nosocomial and external infections (Naghavi et al., 2024). Biocidal products, on the other hand, exert microbiostatic or microbiocidal effects against a broad range of micro-organisms, and some of them can facilitate the selection of mutations that confer antibiotic resistance; additionally, they can select for antibiotic resistance in microbial pathogens by resistance or cross-resistance mechanisms (Larsson and Flach, 2022; Wales and Davies, 2015). The presence of metal(loid)s in the environment—both natural and due to anthropogenic activities – can play an important role in the maintaining and triggering the proliferation of ARGs (Baker-Austin et al., 2006; Hu et al., 2017; Ji et al., 2012; Knapp et al., 2011; Safari Sinegani and Younessi, 2017; Zhao et al., 2019; Zheng et al., 2022). Interplay between metal(loid)s and antimicrobial resistance includes co-resistance (co-selection) for both resistance types (with two or more resistance genes linked physically, e.g., by being propagated via the same mobile genetic elements), cross-resistance (metal(loid) and antibiotic resistance genes linked functionally, e.g. non-specific multi-drug efflux pumps), and co-regulation, where complex coordinated transcriptional and translational bacterial responses to the stress introduced by reactive oxygen forms and metal(loid)s leads to increased resistance to antimicrobial compounds (Baker-Austin et al., 2006; Bobate et al., 2023; Gillieatt and Coleman, 2024; Qiu et al., 2023; Teixeira et al., 2016; Wang et al., 2021; Xu and Lin, 2024; Zhao et al., 2019). The co-selection mechanism enables the selection of antibiotic resistance genes in the presence of metal(loid)s in antibiotic-free environments, although the molecular aspects of these phenomena are not well defined, nor are the specific influences of different environmental variables and to what extent the environment contributes to the evolution of antimicrobial resistance (Gillieatt and Coleman, 2024). Co-selection phenomena of antibiotic and metal(loid) resistance have been described for arsenic (As), cadmium (Cd), cobalt (Co), chromium (Cr), copper (Cu), mercury (Hg), nickel (Ni), lead (Pb), and zinc (Zn), and their presence in urban soil was found to increase the potential for the horizontal gene transfer of ARGs and metal(loid) resistance genes (MRGs) by integrons and transposons (Zhao et al., 2019, 2024). Pb, Zn and Cu were found to promote conjugative HGT rates of ARGs in bacteria in freshwater microcosms (Wang et al., 2020a), and Cu was capable of increasing the exchange of antibiotic resistance genes between environmental and pathogenic bacteria in clinical environments (Virieux-Petit et al., 2022), as well as influencing the abundances of gut-associated ARGs and MRGs in fly larvae (Wu et al., 2021). One important and often-overlooked aspect is that, rather than the mere presence of metal(loid)s or biocides, the most determining factor in the co-occurrence of metal(loid) and biocide resistance genes and antibiotic resistance genes (ARGs) in plasmids was the historical exposure of microbial communities to antibiotics and antibiotic-related selective pressures (Pal et al., 2017). In fact, the is ample evidence that correlation between the spread of antimicrobial and metal(loid) resistance is noticeably higher in bacteria inhabiting the sites with a significant influx of both antibiotics and metal(loid)s, such as landfills, animal farms, agricultural land and urban soils (Guo et al., 2025; Gupta et al., 2022; Mazhar et al., 2021; Mokni-Tlili et al., 2023; Wang et al., 2020b; Zhang et al., 2021; Zhao et al., 2020, 2019). Study of co-occurrence in natural environments, or contaminated environments that lacked antibiotic input is not as common; however, in one genome-level study measuring co-occurrence of ARGs and MRGs as a function of distance between them in the genomes, this co-occurrence was significantly less frequent in bacteria that were not commonly associated with humans (that suffer both antibiotic and metal(loid) exposure), such as non-pathogenic bacteria inhabiting soil and water (Li et al., 2017). However, the presence of metal(loid)s and biocides seemed to have an important role in maintaining the co-resistance in strains that have already developed this capability (Pal et al., 2017, 2015).

In a previous study (Prosenkov et al., 2023), we explored taxonomic and functional diversity of prokaryotic and eukaryotic microbial communities in diverse environments of El Terronal brownfield (Asturias, Spain) using metagenomic techniques. This former mercury mining and metallurgy site has exceptionally high levels of arsenic and mercury contamination, in addition to a discernible amount of organic contaminants such as polycyclic aromatic hydrocarbons (PAHs) and phenylmercury propionate (Gallego et al., 2015; Loredo et al., 2003). Bacteria known to carry mercury resistance determinants from the phyla Proteobacteria, Firmicutes, and Actinobacteria were abundantly represented, although microbial diversity decreased sharply in the most contaminated pyrometallurgical waste compared to the surrounding soils that were contaminated to a lesser degree. Functional metagenome predictions carried out in that study showed the probable presence of mercury and arsenic resistance/detoxification genes, which tended to be more abundant in the presence of higher concentrations of those metal(oids). In the present work we have significantly extended the previous analysis of El Terronal microbial communities by isolating, culturing, and characterizing a large collection of bacterial strains from contaminated soils, groundwater, groundwater sediments and pyrometallurgical waste from the site. Availability of these bacteria in culture greatly facilitates the investigation of co-selection mechanisms for resistance determinants to these metal(loid)s and antibiotics in such a highly contaminated environment, and allows for the evaluation of the hypothetical health risk of the site. As already suggested by other authors (Gillieatt and Coleman, 2024), the study of bacteria in environments highly contaminated with metal(loid)s that had only minimal co-contamination with antibiotics would help to study their role as environmental reservoirs of ARGs, and determine possible transmission mechanisms. El Terronal site was developed intermittently since the times of the Roman Empire, reaching its production peak in the 1960s when it became Spain's second-largest (and the world's 8th-largest) mercury producer, with annual output of up to 15,000 flasks (~517.5 tons) of mercury; however, it had no human activity since the factory's closure in the 1973. Its facilities were either mothballed or abandoned entirely, and access to the site was restricted. It is therefore an ideal model for analyzing evolutionary pressure caused by metal(loid) contamination—including the degree to which antibiotic resistance was favored and propagated by the acquisition of resistance to metal(loid)s in an environment without any known influx of antimicrobial substances of human origin.

## 2 Materials and methods

### 2.1 Sampling

**Figure 1:**
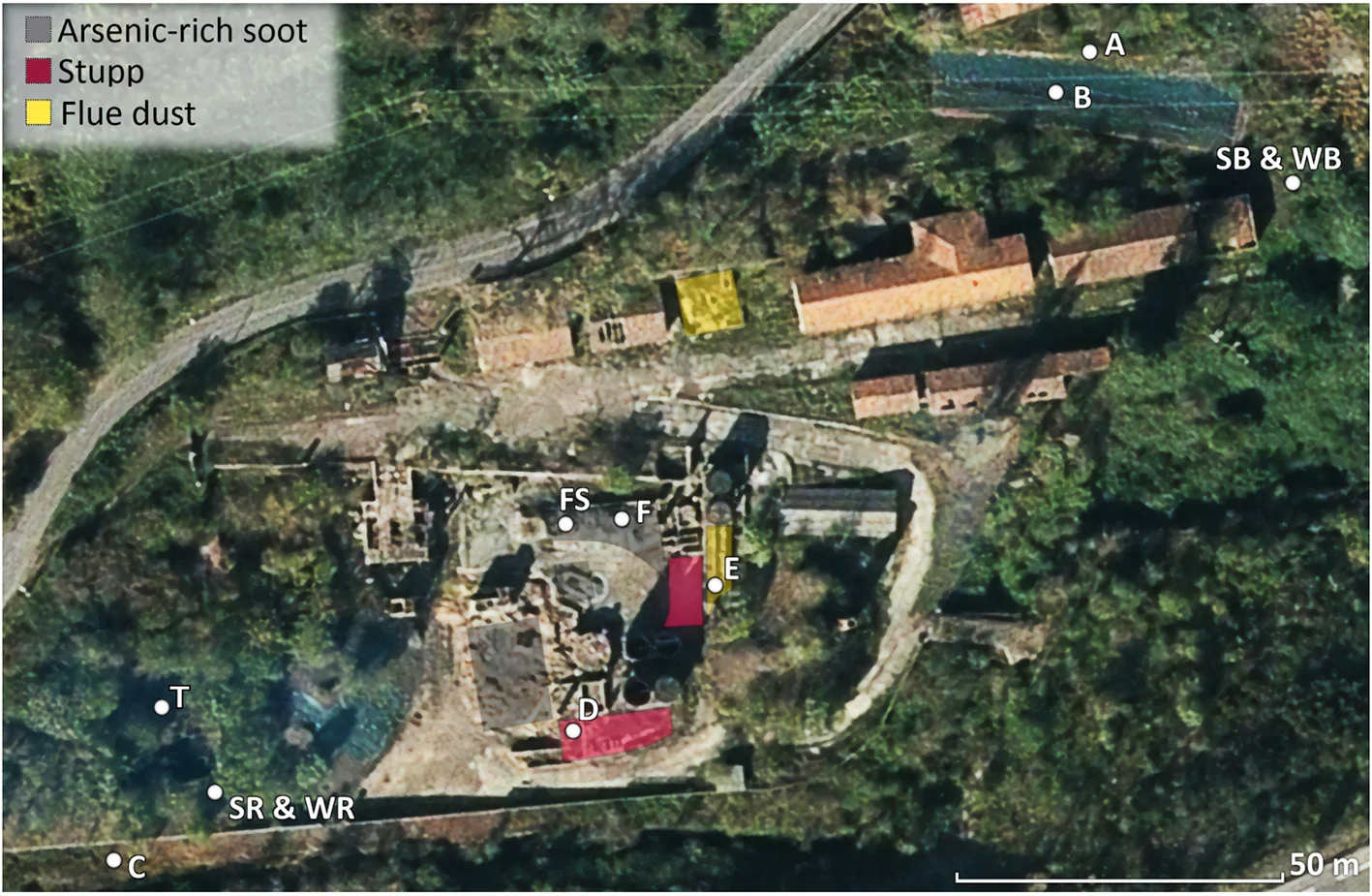
Sampling locations of the different types of pyro metallurgical waste. Satellite imagery obtained via ArcGIS web server using World Imagery map layer with subsequent upscaling. Sample types: soil (A, B, C & T), pyrometallurgical waste (D, E, F & FS), groundwater (WB & WR) and groundwater sediments (SB & SR). Map sources: Esri Community Maps Contributors, Dirección General de Catastro, CC-BY 4.0 scne.es, Esri, HERE, Garmin, INCREMENT P, METI/NASA, USGS.

Several sampling campaigns took place, with each campaign extending the coverage to new environments within the site. Contaminated soils, as well as various types of pyrometallurgical waste (As-rich soot, stupp and flue dust) located at the defunct industrial installations were sampled in 2015 (samples A–F). Groundwater and groundwater sediments, as well as additional sample of As-rich soot were taken in 2016 (sample FS, SB, SR, WB and WR); and additional sample of contaminated soil from a wooded area within the site's premises (sample T) was taken in 2020 (Figure 1). The pyrometallurgical production process and the mineralogical and chemical characteristics of these residues/samples have been described in detail previously (Gallego et al., 2015; González-Fernández et al., 2018; Prosenkov et al., 2023). Sampled materials were placed into sterile 50 mL plastic tubes (Labbox, Spain) and processed within 2 days. To determine arsenic and mercury concentration in solids, representative subsamples of soils, waste and groundwater sediments were dried, ground to the particle size of less than 100 μm and digested in ‘*aqua regia*' (HCl + HNO_3_, 3:1) in an Anton Paar 3,000 microwave. As and Hg were quantified by Inductively Coupled Plasma Mass Spectrometry (ICP-MS 7700, Agilent Technologies, California, USA) using IDA (Isotopic Dilution Analysis) with a spike solution from ISC Science, Spain. High-purity standards (Charleston, SC, USA) for calibration and a Certified Reference Material (soil, ERM-CC018) were used.

### 2.2 Isolation and identification of bacterial strains

For isolation of bacteria, 5 g of sampled material were resuspended in 10 mL of 0.1M sodium pyrophosphate (Na_4_P_2_O_7_), agitated on a FV15013 Vortex shaker (Fisher Scientific, USA) for 10 min, and allowed to settle for 5 min. After that, 1 mL of supernatant was taken from each sample, and used to make serial dilutions of up to 10^−7^ for seeding on 1/10 Tryptic Soy Agar (TSA, Sigma-Aldrich, USA) plates. Groundwater samples were seeded directly. Agar plates were cultivated for 2 days at 28 °C, after which morphologically distinct individual colonies were picked with a sterile seeding hook and streaked onto 1/10 TSA plates to obtain pure cultures. Resulting collection of strains was preserved by freezing in 10% skim milk (Oxoid, UK), as well as by freeze-drying in 20% skim milk and 1.7% trehalose (Oxoid, UK).

To identify those strains, their DNA was extracted using GeneMATRIX Bacterial and Yeast Genomic DNA Purification Kit (EURx, Poland). 16S rRNA genes were amplified by PCR using OptiTac polymerase (EURx) with primers 27F and 1492R (Lane, 1991), purified using Illustra GFX PCR DNA and Gel Band Purification kit (GE Healthcare, USA), and sequenced by Sanger sequencing at Scientific-Technological Services of the University of Oviedo, Spain, using standard bacterial 16S rRNA primers 27F, 907R, 1492R (Lane, 1991) and 341F (Muyzer et al., 1993). Complete sequences of 16S rRNA gene were reconstructed using Ugene (Okonechnikov et al., 2012). Identification was performed against Silva 138 database (Yilmaz et al., 2014).

### 2.3 Determination of As and Hg resistance

The terms “tolerance” and “resistance” as used in clinical environment are not applicable to metal(loid)s since there are no predetermined breakpoints or MICs (Wales and Davies, 2015). Here, being “resistant” to a given concentration of metal(loid) refers to the highest concentration at which growth was observed. To that end, all isolated strains were seeded on 1/10 TSA plates supplemented with increasing concentrations of either As(III) (as sodium arsenite; VWR, USA), As(V) (as sodium arsenate; Sigma-Aldrich, USA) or mercury(II) (as mercury(II) chloride; Sigma-Aldrich, USA) using a sterile toothpick, and incubated for 24 h at 28°C, followed by 48 hours at room temperature to account for strains that exhibited slower growth at elevated temperatures (Table 1). All these compounds had been detected at the polluted site (Gallego et al., 2015; Rumayor et al., 2017); concentration ranges were selected based on previous studies of hyper-resistant strains isolated from contaminated environments (Bermanec et al., 2021; Ruiz-Díez et al., 2012), as well as our results in previous experiments (unpublished data). Plates were visually inspected for signs of bacterial growth after 24 h and after 48 h of incubation, and the highest concentrations of contaminants at which each strain exhibited growth were recorded.

**Table 1:** Concentrations of NaAsO_2_, Na_2_HAsO_4_ and HgCl_2_ used for characterization of As(III), As(V) and Hg(II) resistance.

Contaminant	Concentrations					
As(III)	2 mM	5 mM	10 mM	15 mM	20 mM	25 mM
As(V)	5 mM	20 mM	50 mM	100 mM	200 mM	300 mM
Hg(II)	25 μM	50 μM	100 μM	200 μM	300 μM	400 μM

### 2.4 Antimicrobial susceptibility testing

Antimicrobial susceptibility to antibiotics and biocides was screened using disk diffusion method following a modification of the most current EUCAST susceptibility testing guidelines (EUCAST, 2017). Briefly, a single colony was used to prepare a bacterial suspension to McFarland 0.5 turbidity standard (REMEL, ThermoFisher Scientific, UK) for each strain, which was used to inoculate Mueller-Hinton agar (Oxoid, UK) plates using sterile cotton swabs. Disks impregnated with antimicrobial agents (Table 2) were then placed on the surface of agar. Antibiotic disks were sourced from commercial suppliers; disks impregnated with biocides most commonly used in Spain (ethanol, povidone-iodine, chlorhexidine, and sodium hypochlorite; sourced from commercial suppliers) were prepared by impregnating filter paper with 40 μl of each compound. Plates were incubated overnight at 35°C as per EUCAST protocol, and then for additional 24 h at room temperature to account for strains showing limited growth at 35°C. Inoculated plates without antimicrobial compounds were used as growth control; the few strains that were incapable of growth on MH agar were tested on 1:10 TSA instead. *Escherichia coli* ATCC 25922 (wild-type, susceptible to a wide range of antimicrobials) was used for method quality control (Miller et al., 2003), both when testing on MH and 1:10 TSA plates.

**Table 2:** Antibiotics and biocides used for antimicrobial susceptibility testing.

Compound	Code	Dose	Supplier
Antibiotics			
Cefuroxime	CXM	30 μg	Bio-Rad, UK
Erythromycin	E	15 μg	
Ciprofloxacin	CIP	5 μg	
Amoxicillin/Clavulanic Acid	AUG	20/10 μg	
Vancomycin	VAN	30 μg	
Tetracycline	TE	30 μg	
Compound Sulphonamides	S3	300 μg	Oxoid, UK
Trimethoprim	W	5 μg	
Streptomycin	S	10 μg	
Compound	Code	Dose (effective)	Supplier
Biocides			
Ethanol	ET	55,25 mg	Acofarma Distribución S.A., Spain
Povidone-iodine	PI	4 mg	
Chlorhexidine	CH	800 μg	Lainco, S.A., Spain
Sodium hypochlorite	SH	1.4 mg	Henkel Ibérica, S.A., Spain

Growth inhibition zones were measured with a ruler, with plates held against a dark background. In absence of any widely recognized guidelines for antibiotic susceptibility testing of environmental strains, a highly conservative criteria were chosen, where only the complete lack of inhibition zone around the disk was interpreted as indicative of resistance. Vancomycin data was adjusted to account for its narrow spectrum using taxonomic identification data, with Gram-negative bacteria deemed naturally resistant due to the lack of target for antibiotic action. In addition to this conservative interpretation, criteria available in the most current version (v.15.0) of EUCAST Clinical Breakpoints table (EUCAST, 2025) were used for *Pseudomonas* strains when testing for resistance to ciprofloxacin, and for *Bacillus* strains when testing for resistance to erythromycin, ciprofloxacin and vancomycin.

### 2.5 Statistical analysis

Multiple Antibiotic Resistance (MAR) index was calculated as the ratio between the number of antibiotics that given strain was resistant to and the total number of antibiotics tested (Krumperman, 1983). Data was adjusted to account for the narrow spectrum of vancomycin, excluding it from MAR calculation for Gram-negative strains.

Correlation between resistance to various antibiotics and biocides and resistance to arsenic and mercury was studied both on raw data (diameter of the zone of growth inhibition for each antimicrobial compound tested) and interpreted data (strains deemed Resistant or Susceptible) using Kendall's τ (Kendall, 1938) and logistic regression, respectively. Logistic regression models were built independently for each antibiotic-metal(loid) pair; null hypothesis assumed relationship between metal(loid) resistance (predictor variable) and antibiotic resistance (dependent variable). Wald test was used to check for statistical significance. All calculations were performed in R (R Core Team, 2021) with the help of packages *psych* and *nnet*. Data was adjusted to account for the narrow spectrum of vancomycin, excluding it from both raw data and interpreted results for Gram-negative strains.

### 2.6 Genome sequencing, assembly and annotation

Some of the isolated strains were selected for genome sequencing, based on their high resistance both to arsenic and/or mercury and antibiotics. Their DNA was extracted using NZY Microbial gDNA Isolation kit (NZYTech, Portugal); 2 × 150 bp Illumina HiSeq sequencing was performed by Eurofins Genomics (Germany).

SKEWER (Jiang et al., 2014) was used for adapter trimming and quality filtering and FLASH (Magoč and Salzberg, 2011) was used for joining overlapping reads. Assembly was performed using SPAdes v3.13.1 (Bankevich et al., 2012), with subsequent quality control using Bandage (Wick et al., 2015) and QUAST (Gurevich et al., 2013). To assess possible contamination, all contigs produced by SPAdes were imported into UGENE and taxonomically classified by KRAKEN (Wood and Salzberg, 2014) CLARK (Ounit et al., 2015), and DIAMOND (Buchfink et al., 2015), with subsequent consensus classification generated by WEVOTE (Metwally et al., 2016). Taxonomic classification, as well as contigs and coverage information produced by SPAdes were imported into BlobTools (Laetsch and Blaxter, 2017) for visualization and filtering, where contigs that showed highly dissimilar coverage, GC skew and taxonomic identity compared to the majority of contigs were removed. Scaffolding with ScaffMatch (Mandric and Zelikovsky, 2015) was performed on remaining contigs, with subsequent gap filling with GapFiller (Boetzer and Pirovano, 2012) and final polishing with ntEdit (Warren et al., 2019). Additionally, plasmid assembly was performed with plasmidSPAdes (Antipov et al., 2016) with subsequent polishing with ntEdit.

Similarity between genomes of strains belonging to the same genus was evaluated by synteny analysis. Synteny blocks are non-overlapping, highly conserved segments any given genome can be decomposed into; the number of shared synteny blocks between two or more genomes, and proportion of each genome represented by them are indicative of how related organisms are to each other. Decomposition into synteny blocks was performed using Sibelia (Minkin et al., 2013 with minimal synteny block size of 2500 nucleotides. Visualization was performed in Circos (Krzywinski et al., 2009). Genome-level identification was performed by estimating Average Nucleotide Identity (ANI) against genomes from GTDB-r226 database with skani (Shaw and Yu, 2023), using 95% cut-off for species demarcation (Jain et al., 2018).

Draft genomes were annotated using BAKTA (Schwengers et al., 2021) for general annotation using full version of bakta database; this included annotating arsenic and mercury resistance genes. RGI-CARD (Alcock et al., 2023) was used for antibiotic resistance genes annotation, with options for Perfect and Strict hits (with *nudged hits* option for inclusion of borderline results), and less restrictive search (loose hits) against sequences of integrative and conjugative elements in the genome retrieved using ICEfinder front-end for the ICEberg 2.0 database (Liu et al., 2019) andmobileOG-db annotations (Brown et al., 2022) that were used for finding all putative mobile elements within the genomes. Additionally, (Vernikos and Parkhill 2006) was used for evidence of putative horizontal gene transfer (HGT) events. Annotated genomes including arsenic and mercury resistance genes, antibiotic resistance genes, MGE genes such as transposon genes, integrase genes and cassettes, prophages, etc., as well as putative HGT regions were visualized and analyzed manually using Proksee web server (Grant et al., 2023). Putative MGE regions were identified on the basis of presence of MGE-specific genes and genomic elements (integrases, transposases, integron cassettes, etc.) and inspected for presence of arsenic, mercury and antibiotic resistance genes, and enumerated accordingly. Additional visualizations were created with MATLAB and GenomeTools (Gremme et al., 2013).

## 3 Results and discussion

### 3.1 Sample collection and characterization

As already highlighted in the previous work (Prosenkov et al., 2023), pyrometallurgical waste has shown the highest level of arsenic contamination, with arsenic-rich soot (F, FS samples) in demolished arsenic condensers containing around 55% of arsenic and almost 3% of mercury. Stupp (sample D)—a residue found in mercury condensers at the site—had lower concentration of arsenic (around 12%), but much higher concentration of mercury, almost reaching 6.6%. Flue dust (sample E) was about 2% arsenic and 0.77% mercury by weight. Topsoil (sample A), mixed and tilled soil (sample B) from the plot attached to the industrial site where a phytoremediation trial was previously carried out (Mesa et al., 2017), soil that formed naturally over time on top of the heavily contaminated riverbank (sample C), soil from the wooded area (T) and groundwater sediments (samples SB and SR) all showed a very high degree of contamination, significantly exceeding fairly permissive safe limits of 200 mg/kg of As and 100 mg/kg of mercury allowed by local legislation for industrial land use (BOPA, 2014). The relevant chemical characteristics of the different samples studied (Prosenkov et al., 2023) are detailed in Supplementary Table 1.

### 3.2 Isolation and identification of bacterial strains

**Figure 2:**
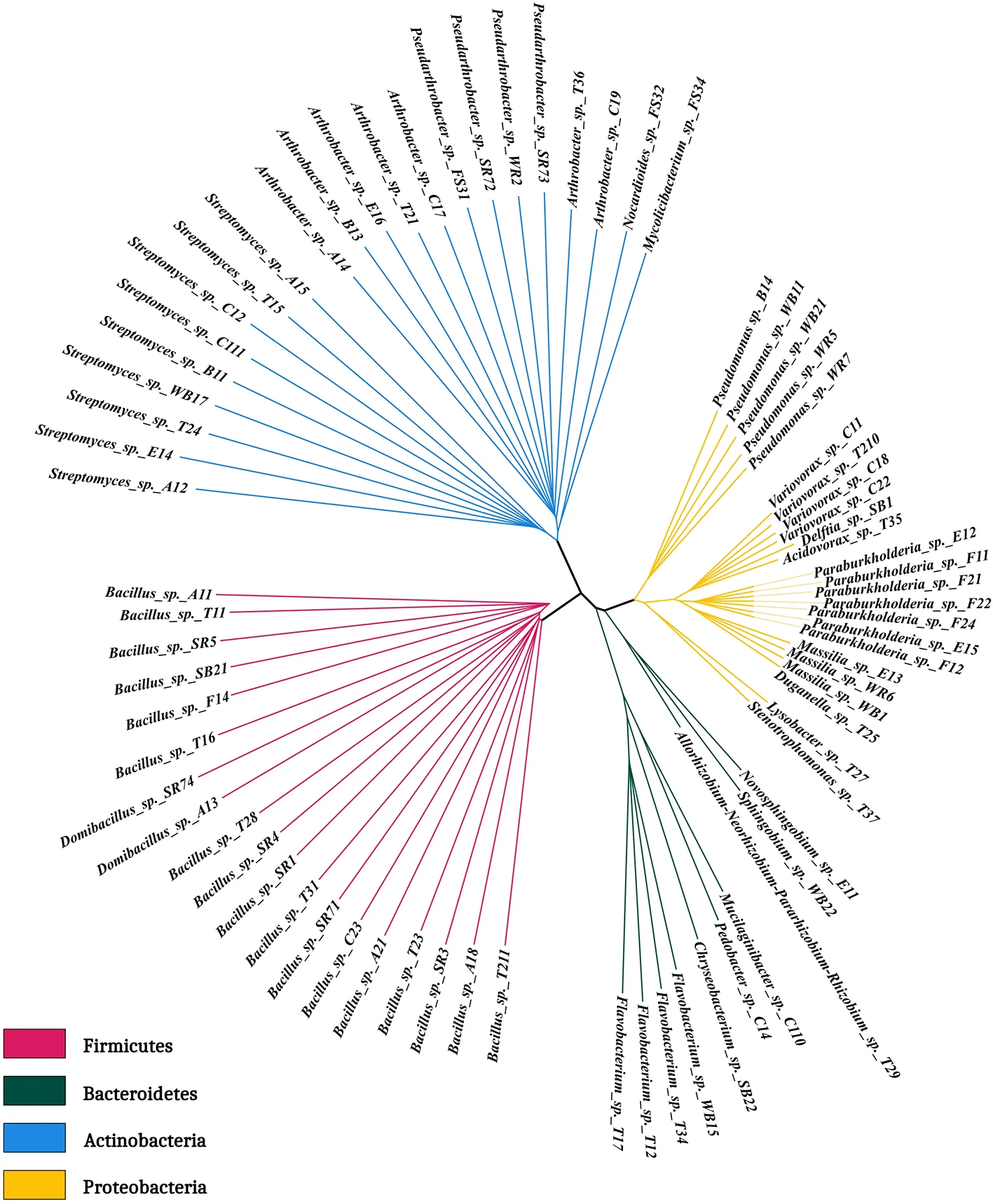
Phylogram of bacterial strains isolated from El Terronal site, with phylum-level taxonomic information.

In the metagenomic analysis of El Terronal brownfield performed previously, microbial communities in soils and pyrometallurgical waste where dominated by Proteobacteria, Actinobacteria, and Acidobacteria, followed by Firmicutes, Bacteroidetes, and others, with a marked decrease in diversity and evenness as well as significant shifts in the composition of microbial communities in the most contaminated samples (Prosenkov et al., 2023). In the present work, we completed and extended this analysis by isolating, culturing, and characterizing a total of 74 bacterial strains from soils, groundwater and groundwater sediments, and different types of pyrometallurgical waste (stupp, flue dust, and As-rich soot). Most of them belonged to the phyla mentioned above (Proteobacteria, Actinobacteria, or Firmicutes), with a small minority belonging to the phylum Bacteroidetes (Figure 2). Members of the phylum Acidobacteria require more demanding isolation and culture conditions (Kalam et al., 2020) and are not represented among strains isolated here. Due to their lower relative abundance and/or difficulties in growing in the laboratory, other phyla described in the metagenomic analysis, such as Chloroflexi, Planctomycetes, and Gemmatimonadetes (Prosenkov et al., 2023), did not produce any cultured representatives in this work. Similarly, but not unexpectedly, the stupp sample yielded no strains capable of growth on 1/10 TSA, a result that corroborates previous metagenomic data which indicated that the overwhelming majority of prokaryotic microorganisms in this residue were mesophyllic archaea of the phylum Crenarchaeota, equally difficult to isolate under the conventional heterotrophic conditions used in the laboratory (Simon et al., 2005).

Strains belonging to the different phyla were obtained from all samples regardless of their origin and level of contamination, but with some peculiarities. As would be expected, the largest number of bacteria of the genus *Streptomyces* came from the soil (Timková et al., 2018), especially from the soil sample taken in the wooded area (see below). Phylum Firmicutes was represented mainly by members of the genus *Bacillus*, known to have high tolerance to metals, and that has the ability to extracellularly sequester Hg through the secretion of negatively charged extracellular polymeric substances (EPS; Mejias Carpio et al., 2017; Priyadarshanee et al., 2022). *Bacillus* were the predominant Gram-positive bacteria isolated from the soil of Minamata Bay (Japan), the Almadén mercury mining district (Ciudad Real, Spain), and other environments contaminated with Hg (Nakamura and Silver, 1994; Robas et al., 2021). Genera *Paraburkholderia, Pseudomonas, Variovorax* and *Massilia* made the bulk of cultured Proteobacteria in El Terronal, and the Actinobacteria strains belonged mostly to genera *Streptomyces, Arthrobacter* and *Pseudarthrobacter*.

The presence of *Massilia spp*. is noteworthy, as they play an important role in detoxification of heavy metals, degradation of organic pollutants, promotion of the plant resistance and absorption of pollutants, making them potentially useful tools in bioremediation and phytoremediation technologies (Amirhosseini et al., 2025). Representatives of the genus *Streptomyces* were found in soils contaminated with As, Hg, Cd, and Mn (Kuramata et al., 2014; Rahayu et al., 2021), although they were absent in other less extreme environments also contaminated with As, Hg or both (Aguilar et al., 2020; Atibu et al., 2023; Niane et al., 2019; Valverde et al., 2011). Their metabolic, structural and growth characteristics are ideal for the bioremediation of metals such as chromium, manganese, nickel, zinc, copper and lead (Reti et al., 2024; Sanjenbam et al., 2012; Timková et al., 2018), these bacteria are also notable producers of antibiotics and other bioactive compounds (Donald et al., 2022).

Representatives of the Bacteroidetes phylum were more diverse (Figure 2): Gram-negative *Pedobacter* bacteria were resistant against numerous antibiotics from several classes (Viana et al., 2018) and, together with bacteria from the genus *Mucilaginibacter* have been linked to plant biomass decomposition (López-Mondéjar et al., 2016). Highly Hg-resistant *Sphingobium* strains have previously been isolated from Hg-contaminated soils (Mahbub et al., 2016); furthermore, they have been linked to the degradation of very recalcitrant organic compounds such as lindane (Khan et al., 2022; Peña-Álvarez et al., 2025). All isolated strains of the genus *Paraburkholderia* came from the most heavily contaminated pyrometallurgical waste samples: flue dust (E) and As-rich soot (F). These bacterial isolates deserve further study, as this genus includes several species with a notable capacity to improve crop yield, inhibit phytopathogens, enhance systemic plant resistance, or reduce heavy metal accumulation (Rojas-Rojas et al., 2025).

Since sampling was done with the intent to maximize the number of sampled environments and increase the number and diversity of isolated strains belonging to different taxonomic groups, only very limited data is available on seasonal differences and species turnover between sampling times. The only environment that was sampled twice is As-rich soot (samples F and FS). The differences in metagenomic profiles of microbial communities there were analyzed in detail previously (Prosenkov et al., 2023); there, samples F and FS were found to harbor a small core community consisting of *Paraburkholderia, Acidocella* and *Bacillus* that persisted between samplings, with sample F dominated by *Paraburkholderia spp*., and sample FS—by bacteria belonging genera *Acidiphilium* (of Alphaproteobacteria) and *Chryseobacterium* (Bacteroidetes). In the current study, strains isolated from As-rich soot in the winter of 2015 (sample F) belonged mainly to the Proteobacteria of the genus *Paraburkholderia* and *Bacillus*, thus being representative of the wider microbial community, while all strains isolated in the summer of 2016 belonged to Actinobacteria. It is very likely that this difference is due to seasonal variation in environmental conditions, with higher humidity in the winter allowing expansion of *Paraburkholderia* strains and their significantly higher isolation rate.

### 3.3 Arsenic, mercury, antibiotic and biocide resistance of isolated strains

Majority of isolated strains had at least some level of resistance to either As(III), As(V) or HgCl_2_ (Supplementary Table 2). As would be expected, bacteria isolated from As-rich soot (F, FS) and groundwater sediments were generally more resistant, while strains with lowest level of resistance to those metal(loid)s were isolated from less-contaminated soils and groundwater; several hyper-resistant strains capable of surviving up to 20 mM of As(III) and 400 μM of HgCl_2_ were isolated from those environments as well. Interestingly, soil sample T provided the highest number of strains with low resistance to mercury, despite having high levels of mercury contamination; it might be related to its high organic carbon content, exceeding that of other sampled soils. It is thought that high amounts of organic carbon can lower mercury bioavailability due to its bonding to humic acids (Lee et al., 2023; Różański et al., 2016).

Resistance to antimicrobial compounds is a concept derived from their application in human and veterinary medicine. In clinical practice, resistance and susceptibility of a given strain is determined based largely on empirical data and is frequently changed and adjusted as resistance profiles of bacteria change or more data become available. It is governed by a decision-making rather than data-gathering framework and aimed at characterizing known infectious agents and selecting appropriate therapeutic strategies (CLSI, 2024; Matuschek et al., 2014). Since they are not usually pathogenic and are not defined by clinical breakpoint concentrations, resistance in environmental microorganisms typically refers to a lower susceptibility to an antibiotic compared to other strains of the same species (Larsson and Flach, 2022). However, due to the lack of widely established guidelines and data interpretation criteria, susceptibility and resistance to antimicrobial compounds are very difficult to quantify. One approach relies on obtaining isolates in culture media with addition of antibiotics and an arbitrary antibiotic concentration cut-off and subsequent molecular study of genetic determinants of antibiotic resistance to avoid the problem of data interpretation (D'Costa et al., 2006; Osbiston et al., 2021; Pawlowski et al., 2016; Walsh and Duffy, 2013). An alternative option leverages well-established techniques from clinical practice for search of antibiotic-resistant bacterial strains. Determining minimal inhibitory concentration for a given antibiotic was done in several studies and performing antibiotic inactivation assays can further refine the search for antibiotic resistance (Gattinger et al., 2023; Pawlowski et al., 2016). Disk diffusion test is another clinical method that allows for rapid screening of big collections of isolates without the need to establish an enrichment culture. The problem of data interpretation (determining which strains are resistant and which are not) is usually solved by either extending available guidelines to environmental strains (Jardine et al., 2019; Safari Sinegani and Younessi, 2017), relying on comparison with control strains (Paun et al., 2021; Pawlowski et al., 2016), or by using an average of breakpoints taken from the resistance data established by EUCAST and CLSI (Gattinger et al., 2023). While the latter practice, in some form, was common even in the clinical environment (in the form of “Epidemiological Cut-Off Values,” derived from distributions of MIC for susceptible and resistant strains), it is now heavily discouraged by EUCAST, at least, in epidemiological context (Kahlmeter and Turnidge, 2022).

For this study, very conservative criteria were chosen, where isolates were considered resistant only when there was no growth inhibition zone around the disc impregnated with antimicrobial compound. While this approach avoids introducing arbitrary cut-off values and eliminates false positives, it does so at the cost of classifying some strains with small no-growth zone diameters (that would have been considered resistant under less restrictive approaches) as susceptible. Under those criteria, 37 out of 74 isolated strains had resistance to at least one antibiotic, and 28 strains were resistant to at least one biocide; 23 strains were sensitive to all the antibiotics and biocides tested (Supplementary Tables 3, 4). Strains *Variovorax sp*. C18 and *Rhizobium* sp. T29 were incapable of growth on MH agar and were tested on 1:10 TSA instead. *Escherichia coli* ATCC 25922 was used as positive control on this media as well; and while it has been long established that culture media influences disk diffusion test results (Brenner and Sherris, 1972), the differences observed with the control strain were small (within 5 mm), and it was considered that strict interpretation criteria that recognized only the absence of no-growth zone as indicative of resistance would negate them.

Two most-resistant strains were isolated from the contaminated soil sample C: *Mucilaginibacter sp*. C110 with MAR index of 0.75 and *Variovorax sp*. C22 with MAR index of 0.625. *Pseudomonas* strains B14, WR7 and WB11 were resistant to half of the antibiotics tested. Unsurprisingly, all isolated *Streptomyces* strains were resistant to at least one antibiotic, with all of them having resistance to trimethoprim. Susceptibility data for vancomycin was adjusted to account for its narrower spectrum; all Gram-negative strains were considered intrinsically resistant due to vancomycin's inability to access their cell walls (Shlaes et al., 1989), and their vancomycin resistance results were discarded from the study. It must be noted that, despite its narrow spectrum, vancomycin has shown at least some growth inhibition effect on most Gram-negative Bacteroidetes bacteria in this study with the exception of *Flavobacterium spp*. T12 and T17 and *Mucilaginibacter sp*. C110. This effect ranged from very limited (no-growth zone d < 10 mm) in case of *Flavobacterium spp*. WB15 and T34 to severe (no-growth zone *d* ≥ 30 mm) in *Novosphingobium sp*. E11, *Sphingobium sp*. WB22, *Rhizobium sp*. T29 and *Pedobacter sp*. C14. Some Proteobacteria strains, especially *Massilia spp*., were showing some growth inhibition as well. Deleterious effect of vancomycin on Bacteroidetes bacteria observed here is in line with intestinal microbial community changes observed in patients undergoing prolonged treatment with vancomycin (Louie et al., 2015), and decrease in intestinal microbiota biodiversity largely occurring at the expense of Bacteroidetes in animal experiments (Isaac et al., 2017). The effect on these bacteria has been further corroborated with animal models in minibioreactors (Auchtung et al., 2025). The mechanism of action of vancomycin on those strains remains poorly understood, and no guidelines are available for susceptibility testing.

Correlations between resistance to As and Hg and diameters of no-growth zones around disks with antibiotics and biocides were either non-existent or weak and not statistically significant, except for a weak negative correlation between resistance to mercury and a no-growth zone diameter for chlorhexidine (Kendall's τ = −0.28, *p* = 0.0163). Chlorhexidine resistance is poorly studied even in clinically relevant strains; however, it is suspected that it relies on non-specific efflux pumps and can result in cross-resistance with other antimicrobial compounds (Abbood et al., 2023) and references therein). In our study, however, the size of the no-growth zone for chlorhexidine was correlated only to the diameter of the no-growth zone for 70% ethanol (Kendall's τ = 0.63, *p* = 2.252E-09). Those findings, while statistically significant and not contradicting possible resistance mechanisms such as action of non-specific multi-drug efflux pumps, are somewhat negated by the lack of corresponding correlations in interpreted data. In fact, it is noteworthy that in the analysis of the resistance data processed according to the chosen conservative criteria, the number of strains classified as resistant to any of the tested antibiotics and biocides was not significantly higher among strains with higher levels of As and Hg resistance. In other words, for strains isolated in this study, possessing high levels of resistance to arsenic and mercury did not correlate to resistance to antibiotics and/or biocides. In contrast to conservative criteria, when EUCAST guidelines were applied to *Bacillus* and *Pseudomonas* strains, all of them could be classified as “Susceptible, increased exposure” (formerly known as “Intermediate”), with *Bacillus* strain SR5 qualifying as “Resistant” when tested against ciprofloxacin. This is similar to mercury-resistant *Bacillus* strains isolated from the rhizosphere and soil at an abandoned metallurgical plant in the Almadén mining district, Ciudad Real, Spain, with mercury concentrations exceeding 106 μg/kg, showed elevated levels of resistance to cephalosporins and tetracyclines compared to the wild type, suggesting that mercury was co-selecting for resistance (Robas et al., 2021). However, in our study isolation was not biased toward mercury-resistant bacteria (culture media was not supplemented with Hg for isolation of bacteria), allowing to isolate Bacillus strains with low mercury and/or arsenic resistance that nonetheless qualified as I (Intermediate/increased exposure) when tested against ciprofloxacin (Supplementary Tables 2, 5), instead suggesting absence of co-selection at least for some of the studied strains (*Bacillus spp*. A11, T11 and T28). Also following EUCAST guidelines, *Bacillus* strains SR3 and T31 could be classified as resistant to erythromycin (Supplementary Table 5). Similarly to conservative interpretation, there was no statistically significant relationship between metal(loid) and antibiotic resistance, nor there was any statistically significant correlation between MAR index and resistance to metal(loids). Therefore, in the current study, both by interpreting the data conservatively and using the EUCAST guidelines, we have found no firm evidence that the presence of arsenic and mercury in El Terronal has led to the emergence and/or spread of antibiotic resistance that would pose a hypothetical clinical threat among the studied strains.

### 3.4 Genome-level analysis of selected strains

Whereas shotgun metagenomics approaches for studying resistance in the environment allow the detection and quantification of any ARG available in databases, culture-based approaches, when combined with whole-genome sequencing allow evaluation of multidrug resistance and provides useful information about both the phenotype and the genetic context of resistance (Bengtsson-Palme et al., 2017; Larsson and Flach, 2022; McLain et al., 2016). In addition, analyzing mobile elements in bacterial genomes allows to determine whether resistance to metal(loid)s and resistance to antimicrobial compounds are connected on genomic level. For this purpose, genomes of 17 bacterial strains originating from different samples from El Terronal were sequenced (Supplementary Table 6). These strains were selected to avoid phylogenetic bias; many of them but not all, have shown high levels of resistance to arsenic and/or mercury, either alone or in combination with high resistance to antimicrobial compounds (Table 3). Additionally, some of them, such as *Arthrobacter* sp. C19 and *Massilia* sp. WB1, were also included for their ability to degrade and emulsify hydrocarbons (unpublished data).

**Table 3:** Strains selected for genome sequencing.

Strain	As(III)	As(V)	HgCl_2_	Cefuroxime	Erythromycin	Ciprofloxacin	Amoxicillin + Clavulanic Acid	Vancomycin	Tetracycline	Compound Sulphonamides	Trimethoprim	Streptomycin	MAR	Ethanol	Povidone-iodine	Chlorhexidine	Sodium hypochlorite
*Pseudomonas sp. B14*	15	300	300					-					0.5				
*Arthrobacter sp. C19*	20	300	50										0				
*Mucilaginibacter sp. C110*	0	100	0					-					0.75				
*Variovorax sp. C22*	25	300	100					-					0.625				
*Paraburkholderia sp. F12*	10	100	300					-					0				
*Bacillus sp. F14*	25	100	400										0.22				
*Paraburkholderia sp. F21*	20	50	400					-					0.125				
*Nocardioides sp. FS32*	25	300	400										0.22				
*Bacillus sp. SR1*	20	200	400										0				
*Bacillus sp. SR3*	20	200	300										0				
*Delftia sp. SB1*	20	300	50					-					0.25				
*Bacillus sp. SB21*	10	200	200										0.22				
*Chryseobacterium sp. SB22*	10	300	200										0.5				
*Pseudomonas sp. WR7*	10	300	400					-					0.5				
*Massilia sp. WB1*	0	20	200					-					0.25				
*Pseudomonas sp. WB11*	5	300	25					-					0.5				
*Streptomyces sp. WB17*	2	300	50										0.1				

▭ Susceptible ▭ Resistant. Highest tolerated concentrations of sodium arsenite, sodium arsenate and mercury chloride are given for each strain. Absence of a no-growth zone around antibiotic-impregnated disks is interpreted as Resistant (*R*), presence of such a zone is interpreted as Susceptible (*S*). Vancomycin results are excluded for Gram-negative strains.

Genome-level similarity between strains belonging to the same genus was determined via synteny analysis. Of four *Bacillus* strains, SR1 and SR3 have shown very little similarity to strains F14 and SB21, and to each other. In contrast, strains F14 and SB21 were very similar, with 96.23% and 83.66% of their genomes covered by shared synteny blocks, respectively (Supplementary Figure 1). *Paraburkholderia* strains F12 and F21 had significantly lower genome-level similarity, with only 21.05% and 19.35% of their genomes as shared synteny blocks (Supplementary Figure 2). *Pseudomonas* strains B14, WR7 and WB11 had very little similarity to each other (Supplementary Figure 3). Average Nucleotide Identity (ANI) was calculated for all sequenced genomes against genomes in GTDB R-226 database (Supplementary Table 7). Most *Pseudomonas* and *Bacillus* strains were similar to known species. *Bacillus sp*. F14 and SB21 had ANI of ~99%, with known *Bacillus cereus* strains. *Bacillus spp*. SR1 and SR3 were most similar to *Priestia megaterium* strain ISL-13 and *Peribacillus frigoritolerans* strain JCM 11681with ANI of 96.59 and 97.64%, respectively; both species were formerly classified as *Bacillus*. *Pseudomonas spp*. strains B14, WR7 and WB11 were similar to known species (ANI > 95%), as were *Paraburkholderia spp*. F12 and F21, *Variovorax sp*. C22, *Delftia sp*. SB1, and *Chryseobacterium sp*. SB22. However, *Arthrobacter* sp. C19, *Mucilaginibacter sp*. C110, Nocardioides sp. FS32 and *Massilia sp*. WB1 had ANI below 95%. This suggests that those strains could either belong to new, previously unknown species (at least in the case of *Mucilaginibacter sp*. C110 where a complete, circular genome was assembled) or have diverged significantly from their parent species under selective pressures existing at the El Terronal site. While ANI estimation with skani is reported to be not particularly sensitive to genome fragmentation (Shaw and Yu, 2023), obtaining complete, non-fragmented genomes could potentially somewhat improve classification accuracy in the future, especially as genome databases keep expanding.

**Figure 3:**
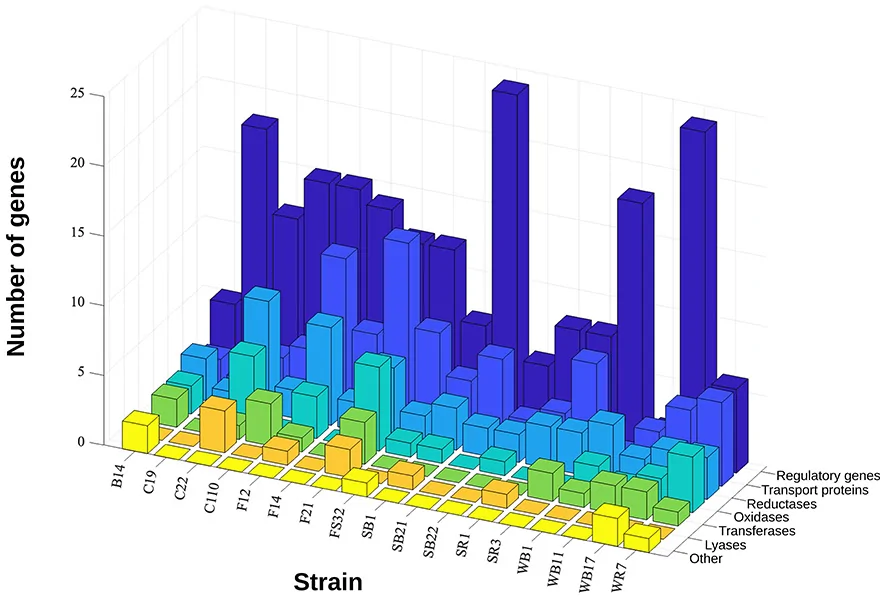
Genes related to arsenic metabolism and detoxification found in sequenced strains, grouped by type.

**Figure 4:**
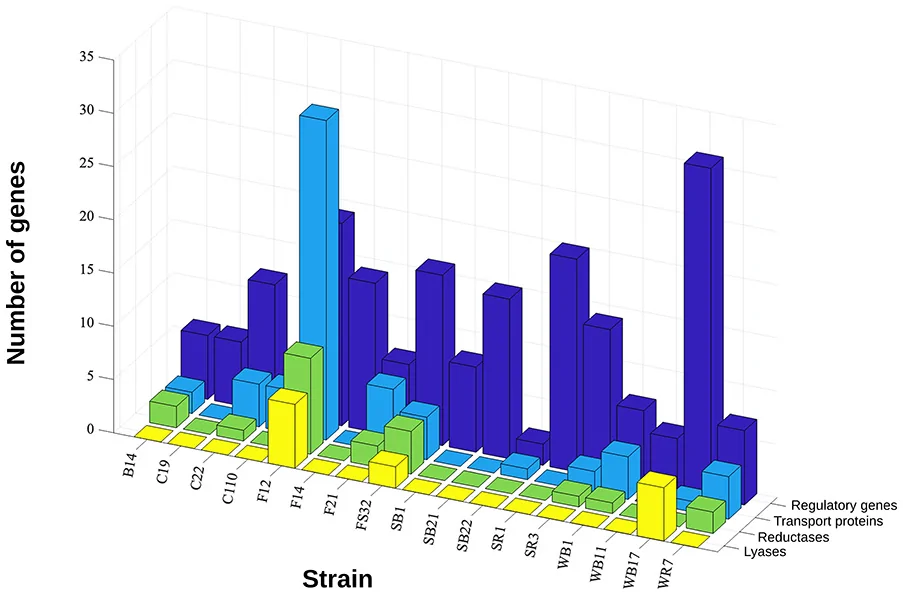
Genes encoding mercury detoxification and resistance found in sequenced strains, grouped by type.

Genome annotation and analysis of all 17 genomes found 403 genes related to As resistance and/or metabolism (Figure 3; Supplementary Table 8). Genes related to As resistance were found both separately and grouped in 31 *ars*-like operons; 4 operons in strains F21 (2 operons), SB1 and SB22 were located in mobile genomic elements, but their completeness could not be evaluated due to gaps in the contigs. Additionally, 289 genes related to Hg resistance were found (Figure 4; Supplementary Table 9), either by themselves or in groups. Seven operons with Hg resistance genes were found, two of which were located in mobile genomic elements, with only one of them, belonging to *Paraburkholderia sp*. F21, appearing intact and functional (Supplementary Figure 4). Seven gene clusters contained both *ars* and *mer* operons; one of them, found in *Variovorax sp*. C22, was located inside a functional mobile element (Supplementary Figure 5). Interestingly, *Arthrobacter* sp. C19 and *Massilia* sp. WB1 had a number of genes related to degradation of complex hydrocarbons, such as biphenyl, naphthalene, benzene and toluene, xylene, and complex, recalcitrant biopolymers such as lignin (data not shown). Those genes and metabolic routes they encode will be analyzed in more detail in future studies.

A total of 114 putative antibiotic resistance genes with high level of similarity to genes with clinical relevance were detected by RGI-CARD (Supplementary Table 10), with 26 of them located inside regions of the chromosome with indications of a putative HGT event (as detected by AlientHunter), suggesting that they could have been acquired through a horizontal transfer event in the past. No instances of genes responsible for arsenic and mercury resistance and antibiotic resistance sharing a mobile genomic element were found. Most of the detected ARGs belonged to different *van* glycopeptide resistance gene clusters (48 genes) and resistance-nodulation-cell division family (RND) of antibiotic efflux pumps, with *adeF* (27 genes) and rsmA (3 genes) being the most common. However, only one of the identified genes—*vanW*, coding for vancomycin B-type resistance protein VanW in *Bacillus sp*. SB21—was located in a mobile element that had a complete set of genes enabling horizontal gene transfer (Supplementary Figure 6). Neither this gene nor other *van* genes from glycopeptide resistance gene clusters found in sequenced genomes seemed to confer any resistance to clinically used concentrations of vancomycin in any of the sequenced strains. In susceptibility testing with disk diffusion method, the no-growth zone was found to be between 18 and 42 mm in diameter, exceeding the 10 mm threshold established for *Bacillus* strains by (EUCAST 2025).

Gene *adeF*, coding for membrane fusion protein of the multidrug efflux complex AdeFGH, was widespread among sequenced strains. Non-specific efflux pumps are ubiquitous in pathogenic bacteria and are among major contributors to antibiotic resistance (Poole, 2004). However, genes coding for other parts of AdeFHG complex could only be found using less restrictive search (“loose hits” in RGI terminology), suggesting an evolutionary history not related to clinical strains.

*BcI* and *BcIII* genes found in *Bacillus spp*. F14 and SB21 were encoding class A β-lactamases of the types I and III, respectively, and thus could be responsible for resistance of those strains to cefuroxime. On the other hand, *Bacillus* strains SR1 and SR3 each had a copy of *BcIII* β-lactamase gene that did not seem to confer cefuroxime resistance. Those two strains also had *qacJ, qacG* (SR1) and *TriB* (SR3) genes encoding small multi-drug efflux pumps that might have been responsible for resistance to some of the biocides tried in this study; however, it is impossible to state with genetic information alone.

*Variovorax sp*. C22 had LRA-1 like β-lactamase; this enzyme was isolated from soil bacteria and was not encountered in clinical isolates, although it could be expressed in *E. coli* and was considered a potential threat if transferred to pathogens (Allen et al., 2009). It could be responsible for the *Variovorax sp. C22* resistance to cefuroxime (Supplementary Tables 3, 4). Gene encoding CGB-1 Ambler-class β-lactamase was found in *Chryseobacterium sp*. SB22; it was originally found in another representative of this genus (Bellais et al., 2002). Strains *Mucilaginibacter sp*. C110, *Variovorax sp*. C22 and *Chryseobacterium sp*. SB22 had very high levels of antibiotic resistance (MAR of 0.71, 0.57 and 0.43, respectively), and strain *Delftia sp*. SB1 was resistant to all four biocides used in this study, but had very few genes with high degree of similarity to known clinically relevant ARGs. However, a large number of putative antibiotic resistance genes was found in the integrative and conjugative elements of their genomes, as well as in genomes of all sequenced strains, even those without detected antibiotic resistance such as *Paraburkholderia sp*. F12 and *Arthrobacter sp*. C19, using less restrictive homology search (Supplementary Figure 7). Some of them could be either non-functional or not confer resistance to clinically used concentrations of antibiotics, but other could be responsible for the high levels of resistance shown by some of the sequenced strains. Determining the hypothetical functions of these genes and their capacity for horizontal gene transfer will be among priority objectives in future studies.

In a previous large-scale study of cultured bacteria from various soils (agricultural sites, urban sites, and pristine environments) it was also determined that high levels of resistance to a wide variety of antibiotics in these bacteria were primarily related to non-specific efflux mechanisms, which could be transferred by mobile genetic elements and were independent of the original soil type (Walsh and Duffy, 2013). It is important to note at this point, that the high levels of intrinsic antibiotic resistance in soil bacteria have not arisen from the same selective pressures as those found in hospital clinical environments; therefore, the corresponding genes are not necessarily related to the function of resistance to these antimicrobials (Alonso et al., 2001; Lee et al., 2010). In other words, clinically relevant soil bacteria do not necessarily possess the same resistance mechanisms as species of the same order isolated, for example, from hospitals, since the evolutionary history of resistance mechanisms in soil bacteria and bacteria operating in the clinical environments are very different (Walsh and Duffy, 2013).

Genetic linkage of mercury or arsenic and antibiotic resistance traits has been documented in some detail (Baker-Austin et al., 2006; Pal et al., 2017; Zhao et al., 2021), which makes the evaluation of MGEs as potential reservoirs and transmission vectors of antibiotic resistance in bacteria even more critical. Analysis performed on public genome databases has shown that the most common metal(loid) resistance gene associations observed in bacterial chromosomes are those for mercury with aminoglycoside, phenicol, sulfonamide, and tetracycline ARGs (Pal et al., 2015), and for arsenic with β-lactam, bacitracin, and fosfomycin ARGs, among others (Gillieatt and Coleman, 2024; Li et al., 2017).

In metagenomic studies of different environments, resistance against aminoglycoside, peptide (vancomycin), tetracycline and β-lactam antibiotics, as well as genes conferring multi-drug resistance were among the most commonly detected. In a study of co-occurrence of ARGs and MRGs in soils contaminated with radionuclides, but lacking any discernable antibiotic influx, multi-drug resistance genes and vancomycin resistance genes were the most abundant (Thomas et al., 2020). In another study performed on poultry farm soils with high influx of (fluoro)quinolones and tetracycline, multi-drug resistance and aminoglycoside resistance genes had the highest abundance (Mazhar et al., 2021). In absence of contamination, as shown by a recent study of ARGs in glacial, ocean and river basin waters (Wang et al., 2024), genes conferring resistance to aminoglycosides, fluoroquinolones, β-lactams, vancomycin, and tetracycline were the most common.

In the current work, only a small fraction of all bacteria inhabiting El Terronal site was analyzed, giving different abundances of ARGs compared to metagenomic studies. Nonetheless, multi-drug, tetracycline and fluoroquinolone resistance genes were the most commonly detected ones, similarly to aforementioned metagenomic studies.

While the presence of arsenic and mercury resistance genes among sequenced strains was ubiquitous, genes responsible for resistance to other metal(loid)s varied greatly between different strains. Presence of genes encoding cadmium-zinc-cobalt resistance proteins of *czc* type was relatively common, but not universal. *Pseudomonas* strains B14 and WR7, despite their genome-level differences, possessed *czcABC*-like operons, *cadA* cadmium efflux pumps and copper resistance operons, while *Pseudomonas sp*. WB11 did not. Operon *czcABC* is known for multidrug resistance dissemination in *Pseudomonas* species, and is frequently associated with antibiotic resistance genes (Cazares et al., 2020); in both *Pseudomonas sp*. B14 and WR7 it was located in mobile genetic elements, and was associated with genes encoding TolC family multi-drug efflux pumps and other RND transporters, including those similar to the *cusA/czcA* family known for conferring copper and silver resistance (Delmar et al., 2015). On the other hand, no cadmium, cobalt, copper, zinc, lead nor nickel resistance genes were detected in the genomes of *Bacillus spp*. F14 and SB21, while *Bacillus sp*. SR1 had efflux pumps for cadmium, cobalt and zinc, and *Bacillus sp*. SR3 had copper resistance genes that were not associated with any putative antibiotic resistance genes nor multi-drug efflux pumps. This shows that, in studied strains, in the absence of strong evolutionary pressure from contamination by other metal(loid)s, retention of corresponding resistance determinants in the genome and their dissemination via HGT may not have happened.

Plasmid assembly yielded only one contig that was positively identified as a part of the plasmid; it was assembled from *Bacillus sp*. SR3 genome, and carried *ars* and *mer* operons, but did not carry any ARGs (Supplementary Figure 8). Contigs assembled from *Paraburkholderia spp*. F12 and F21 were very fragmentary and could not have been reliably linked to plasmid sequences. Our conclusions mirror those from a previous study (Pal et al., 2015) that investigated the occurrence of metal(loid) and antibiotic resistance genes using 2,666 chromosomes and 4,582 plasmids from publicly available databases, revealing only limited potential of metal(loid)s to promote horizontal gene transfer of antibiotic resistance genes, while giving plenty of opportunities to select for antibiotic-resistant bacteria due to widespread presence of metal(loid) resistance genes in the bacterial chromosomes. Despite this, the potential threat from ARGs of the studied bacteria does not appear significant, with no evidence of As and Hg resistance influencing accumulation, persistence and dissemination of ARGs in microbial community. However, examples of strains with very high antimicrobial resistance such as *Mucilaginibacter sp*. C110, *Variovorax sp*. C22 and *Chryseobacterium sp*. SB22, as well as *Delftia sp*. SB1 with its high biocide resistance and no presence of ARGs commonly encountered in clinical isolates shows that those strains, under favorable conditions, could potentially become a source of new antibiotic resistance genes if they could find their way into pathogenic bacteria (Allen et al., 2009). Additionally, the abundance of bacterial resistance mechanisms related to the presence of RND efflux pumps observed in the El Terronal soil bacteria could also enhance the virulence and potential pathogenicity upon transfer to clinically relevant bacteria (Van Allen et al., 2025; Detweiler and Alav, 2025 and references within). This result is consistent with those previously observed by other authors in pot experiments, which showed that Hg contamination is a positive effector of the activation of efflux pumps and bacterial virulence factors (Qiu et al., 2023). Therefore, while the threat from direct dissemination of clinically relevant ARGs might appear minimal, environments highly contaminated with metal(loid)s still constitute a potential reservoir of ARGs, as has already been suggested for the mining region of Almadén (Ciudad Real, Spain), which had similarly high mercury contamination (González et al., 2022).

Although the results of this study are useful and informative, addressing an important question in environmental microbiology, it is necessary to consider that antibiotic resistance in microbial communities is conditioned by many diverse factors in the environment. They include environment type and complexity, diversity and composition of microbial community (Majumdar et al., 2026), as well as presence of antimicrobial substances and other chemical agents (Murray et al., 2024). While El Terronal site had multiple areas contaminated with weathered fuel oil products, we were guided by the previous studies (Gallego et al., 2015) to avoid them during sampling. Therefore, as previously mentioned, the only samples with hydrocarbon contamination were arsenic-rich soot (F), stupp (D) and flue dust (E), where PAHs and organomercurial compounds such as phenylmercury propionate were present. They appeared as a result of incomplete combustion of fuel oil during pyrometallurgic treatment, and preferentially accumulated in flue dust (Gallego et al., 2015). PAHs can act as selective agents for ARGs, especially for resistance determinants that encode aromatic antibiotic efflux pumps, and those mechanisms seem to appear more frequently in Proteobacteria (Chen et al., 2017; Wong et al., 2024). Therefore, we cannot rule out the influence of PAH contamination on microbial communities in As-rich soot and, especially, flue dust; however, microbial community composition of bacteria in the flue dust was more similar to communities in soils than to those in other types of pyrometallurgic waste (Prosenkov et al., 2023), despite high concentration of PAHs there. This is, most likely, due to its chemical and mineralogical composition being more similar to that of the soil (Gallego et al., 2015). Therefore, we consider that PAH contamination present did not alter significantly the results of the study.

Measuring antibiotic resistance in the environment is highly complex and labor-intensive process, and culture-based methods for assessing antibiotic resistance are inherently biased due to the limited representation of the population as a whole by cultured strains (although the real limiting factor is the complexity of the samples themselves; McLain et al., 2016). Also, utilization of the disk diffusion method has less resolving power than determining MIC, and, as mentioned previously, there are no predetermined breakpoints for environmental bacteria nor there are any universal interpretation criteria, which severely limits statistical analysis. Finally, direct study of the genomes to determine relationship between metal(loid) and antibiotic resistance genes is a powerful tool, but short-reads based methods such as Illumina sequencing with subsequent genome assembly tend to produce fragmented genomes and can suffer from assembly errors, leading to incomplete contigs, misassemblies, and inevitable data loss. Detection of ARGs in the genome, especially of the more distant homologs of well-known clinically relevant ARGs does not automatically signify that any given strain has antibiotic resistance. Therefore, for matters as sensitive as ARGs and their mobility within microbial community, both higher-resolution culture-based methods such as MIC and genome sequencing methods capable of producing non-fragmented draft genomes like Nanopore or PacBio sequencing would be advisable. On the other hand, using metagenomic methods can be very useful in determining the presence and abundance of ARGs as it avoids the bias of the strain isolation and cultivation, and, in case of functional metagenomics, allows detection and characterization of novel ARGs (dos Santos et al., 2017). Another possibility that needs to be considered is the presence of alternative non-genetic mechanisms such as production of EPS and growth of biofilms in environmental bacteria, which are known to be relevant metal(loid)-antibiotic co-selection strategies in prokaryotes (Baker-Austin et al., 2006).

## 4 Conclusions

A comprehensive analysis of resistance to arsenic, mercury, and antimicrobial compounds in 74 bacterial strains isolated from soils, groundwater, groundwater sediments, and pyrometallurgical waste in El Terronal did not establish clear patterns of co-occurrence between metal(loid)s resistance and resistance to antibiotics and biocides, suggesting instead a lack of correlation.

Genome sequencing of 17 of the isolated strains revealed a large number of diverse genes related to resistance to arsenic and mercury, many of which were located in functional mobile genomic elements. Most of the detected antibiotic resistance genes, however, were not located in mobile genomic elements, and no genes responsible for metal(loid) resistance and antibiotic resistance were found to share a mobile genomic element. Therefore, any co-occurrence of metal(loid) and antibiotic resistance, at least among strains isolated from the El Terronal site, where high metal(loid) contamination has evolved over time in the absence of anthropogenic antibiotic influx, is likely limited to cross-resistance conferred by non-specific multidrug resistance pumps and, possibly, biofilm formation.

While no firm evidence of the presence of arsenic and mercury influencing the evolution and spread of antibiotic resistance genes among the studied strains was found, the variety and high levels of antimicrobial resistance observed in some of them, and the abundance and diversity of ARGs such as multi-drug efflux pumps observed in the community inhabiting arsenic- and mercury- contaminated soil, justify the need to implement complementary biological containment measures in the management of the site.

## Data Availability

The data supporting the findings of this study are openly available in National Center for Biotechnology Information (NCBI) within the BioProject PRJNA1456026 and under GenBank accession numbers PZ711919–PZ711992.
